# Venetoclax inhibits autophagy in chronic lymphocytic leukemia cells

**DOI:** 10.1080/27694127.2023.2169518

**Published:** 2023-02-07

**Authors:** Yongqiang Chen, Sara E. F. Kost, Xiaoyan Yang, Versha Banerji, James B. Johnston, Sachin Katyal, Spencer B. Gibson

**Affiliations:** a; b Department of Oncology, University of Alberta, Edmonton, Alberta, Canada; c Department of Internal Medicine, Max Rady College of Medicine, Rady Faculty of Health Sciences, University of Manitoba, Winnipeg, Manitoba, Canada; d Department of Biochemistry and Medical Genetics, Max Rady College of Medicine, Rady Faculty of Health, University of Manitoba, Winnipeg, Manitoba, Canada; e Department of Pharmacology and Therapeutics, Max Rady College of Medicine, Rady Faculty of Health University of Manitoba, Winnipeg, Manitoba, Canada; f Department of Immunology, Max Rady College of Medicine, Rady Faculty of Health University of Manitoba, Winnipeg, Manitoba, Canada; g Kipnes Endowed Chair in Lymphatic Disorders, Professor, Department of Oncology, University of Alberta, Edmonton, Alberta, Canada

**Keywords:** Apoptosis, ATG12, autophagic flux, B cells, BCL2, cell survival, chloroquine (CQ), ibrutinib, LC3, CLL, targeted therapy

## Abstract

Venetoclax (VCX) is a BCL2 inhibitor approved for treating B cell-derived leukemia, including chronic lymphocytic leukemia (CLL). VCX’s role in apoptosis induction is well-defined, whereas its other mechanistic roles need to be clarified. Autophagy is an intracellular degradation process involving the lysosome, playing a fundamental role in cancer cell survival and death. In this study, we aim to investigate VCX’s function in autophagy. Following the measurement of autophagic flux by western blot and fluorescent microscopy, we demonstrate that VCX is a novel autophagy inhibitor and reduces the level of the essential autophagy pathway protein ATG12 (autophagy-related 12). VCX sensitizes B cell lines and primary CLL cells to cell death induced by amino acid starvation or ibrutinib. These findings provide a rationale for VCX treatment in combination therapies that inhibit the pro-cell survival function of autophagy in B cell-derived malignancies.

**Abbreviations:** ATG12, autophagy-related 12; BCL2, BCL2 apoptosis regulator; CLL, chronic lymphocytic leukemia; CQ, chloroquine; IBR, ibrutinib; BTK, Bruton’s tyrosine kinase; LC3B, MAP1LC3B (microtubule-associated protein 1 light chain 3 beta); VCX, venetoclax; BECN1, beclin 1

## Introduction

Chronic lymphocytic leukemia (CLL) is the most common form of leukemia in Western countries. It is characterized by the accumulation of monoclonal CD5+ B-lymphocytes (B cells) in the peripheral blood, bone marrow, and lymphoid tissues [1-3]. Current CLL treatments include targeted therapies against B cell receptor (BCR) signaling, such as Bruton’s tyrosine kinase (BTK) inhibitors ibrutinib (IBR) and acalabrutinib, and against antiapoptotic proteins, such as BCL2 which is antagonized by venetoclax (VCX) [1]. VCX is highly effective in CLL and can induce apoptosis in refractory CLL, including those with p53 dysfunction [1,4]. However, the mechanism by which it overcomes drug resistance in CLL is still unclear.

One well-known mechanism for cancer drug resistance is autophagy (macroautophagy). Autophagy is a process of degrading intracellular materials (cargos). It is highly conserved in eukaryotes and induced in response to stresses such as starvation and drug treatment. Autophagy is a dynamic process with multiple sequential steps occurring repeatedly. The canonical autophagy pathway in mammalian cells includes induction, formation of a phagophore with double-membranes, formation of the double-membraned vesicle autophagosome resulting from phagophore expansion, and autolysosome formation through the fusion of the autophagosome with the lysosome. Cargo degradation then occurs in the autolysosome, leading to the generation of small molecules to support cell survival or, under certain conditions, shortage of essential component(s) for cell survival leading to autophagy-induced cell death (AuICD) [5-8].

Due to the dynamic feature of autophagy, functional autophagy activity must be confirmed by measuring autophagic flux. Basal autophagy in primary CLL cells has been demonstrated when autophagic flux was measured by using a lysosomal inhibitor, such as chloroquine (CQ) [9], hydroxychloroquine [10], or bafilomycin[11]. Autophagy (functional autophagy, the same hereafter) can also be induced (at a level higher than that of basal autophagy) by different chemicals in primary CLL cells, including the mammalian target of rapamycin complex 1 (mTORC1) inhibitors rapamycin [12] and KU-0063794 [13], the cyclin-dependent kinase (CDK) inhibitor flavopiridol [12], and the nucleoside analog 8-chloro-adenosine (8-Cl-Ado) [14].

The role of VCX in autophagy has been reported in the breast cancer cell line MDA-MB-231 [15] and a human leukemia monocytic cell line [16]; however, the effect of VCX on autophagy in CLL has not been assessed. The objective of this study is to examine VCX’s roles in autophagic flux and cell death in B cell lines and primary CLL cells. Our research may provide a novel mechanism for VCX treatment of CLL and other cancers.

## Results

### VCX inhibits basal autophagy in B cell lines and primary CLL cells

**Figure 1.**
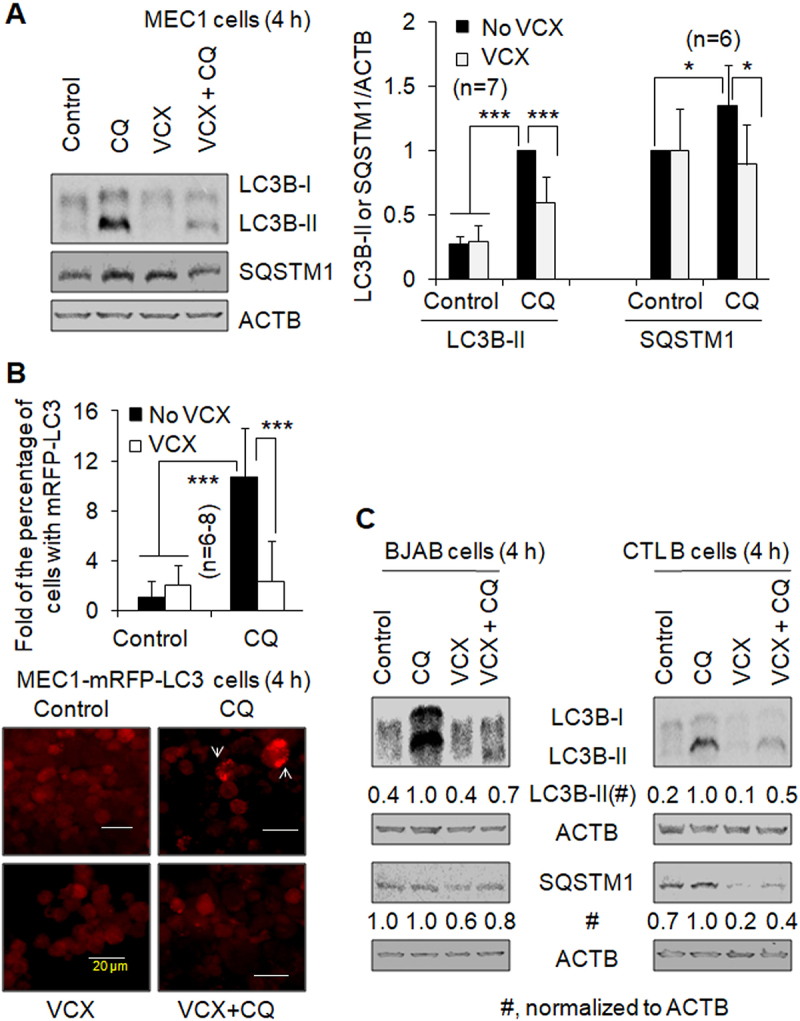
The effect of venetoclax on basal autophagy in B cell lines. Autophagy was measured by western blotting the autophagy marker protein LC3-II (**A, C**) and quantifying the percentage of cells with mRFP-LC3 puncta (**B**) in the absence and presence of the lysosomal inhibitor chloroquine (CQ, 20 µM). SQSTM1/p62, a protein substrate of autophagy, was also measured by western blot. (**A**) Autophagy in MEC1 cells. (**B**) Autophagy in MEC1-mRFP-LC3 cells. Representative images show cells with and without mRFP-LC3 puncta (arrows). At least 30 MEC1-mRFP-LC3 cells were counted per field under a fluorescent microscope. Since the percentage of cells with mRFP-LC3 puncta was less than 10%, the results were demonstrated by “Fold of the percentage of cells with mRFP-LC3 puncta”. (**C**) Autophagy in BJAB cells and the cells of the control normal B cell line (CTL B). VCX, venetoclax (50 nM); * *p*<0.05; *** *p*<0.001. ACTB was used as a loading control.

Autophagy is a stress-response mechanism. Mild stress under a growth or maintenance condition induces basal autophagy in the cell. Basal levels of autophagy promote cell survival and growth [6,8,17]. To investigate the effect of VCX on basal autophagy, we treated B cell lines and primary CLL cells with VCX. Autophagy was evaluated by measuring protein levels of the autophagy marker LC3B-II (the lipidated form of MAP1LC3B (microtubule-associated protein 1 light chain 3 beta)) and the autophagy cargo receptor sequestosome 1 (SQSTM1/p62) and quantify the percentage of cells with mRFP-LC3 puncta, which represent autophagosomes or autolysosomes. The lysosomal inhibitor chloroquine (CQ) was used to assay autophagic flux. A higher level of LC3B-II (or SQSTM1, under some cases) with CQ treatment compared to mock treatment indicates the existence of functional autophagy. In this study, B cell lines, including the human CLL cell line MEC1, the malignant human EBV (Epstein-Bar virus)-negative Burkitt’s-like lymphoma cell line BJAB, an EBV-immortalized normal B cell lymphoblastic cell line (CTL B cells), and primary CLL cells freshly isolated from patients were examined. The addition of CQ increased the LC3B-II protein levels by at least 150% in MEC1, BJAB, and CTL B cells (Fig. 1A, C) and increased the percentage of MEC1 cells with mRFP-LC3 puncta by more than ten folds (Fig. 1B), supporting the concept that functional autophagy occurs in B cell-of-origin lines. In the presence of CQ, 50 nM VCX reduced the LC3B-II protein level by 40% (Fig. 1A) and the percentage of cells with mRFP-LC3 puncta by 78% (Fig. 1B) in MEC1 cells. Furthermore, 50 nM VCX reduced the LC3B-II protein level by 30% in BJAB cells (Fig. 1C) and 50% in CTL B cells (Fig. 1C), respectively, in the presence of CQ. These data indicate that VCX inhibits basal autophagy in B cell lines, supported by the changes of SQSTM1 protein levels in MEC1 and CTL B cells, whereby the addition of CQ increased SQSTM1 protein levels by > 30% and VCX decreased SQSTM1 protein levels in the presence of CQ by > 30% (Fig. 1A, C). In BJAB cells, CQ addition did not change SQSTM1 protein levels, which were reduced by VCX treatment (Fig. 1C).

**Figure 2.**
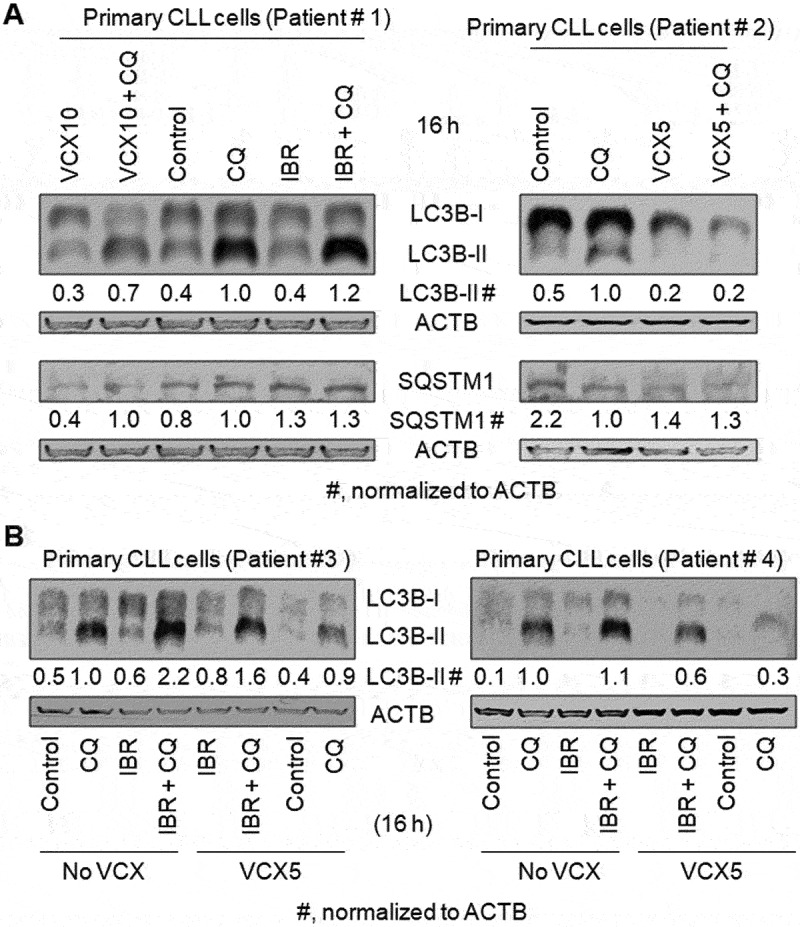
The effect of venetoclax on basal autophagy and ibrutinib-induced autophagy in primary CLL cells. Autophagy was measured by western blotting the autophagy marker protein LC3-II in the absence and presence of the lysosomal inhibitor CQ (20 µM). (**A**) Autophagy in primary CLL cells from Patient #1 and Patient#2. SQSTM1/p62, a protein substrate of autophagy, was also measured by western blot. LC3 western blot was repeated twice with cell lysates of Patient # 1. (**B**) Autophagy in primary CLL cells from Patient #3 and Patient#4. VCX, venetoclax; VCX5, 5 nM VCX; VCX10, 10 nM VCX; IBR, ibrutinib (2.5 µM). ACTB was used as a loading control.

VCX also inhibits basal autophagy in primary CLL cells. The addition of CQ increased LC3B-II protein levels by at least 2-fold in the CLL cells from each of the four patients (Fig. 2). Furthermore, CQ increased the level of SQSTM1 by 25% in the CLL cells from Patient # 1; however, this phenomenon was not observed in the CLL cells from Patient # 2, while VCX did not decrease the relative level of SQSTM1 in the presence of CQ in Patient #1 (Fig. 2A). Combined, VCX inhibits basal autophagy in B cell lines and primary CLL cells, determined by the changes in autophagic flux and SQSTM1 protein levels in a subset of B-cell types.

### VCX inhibits severe stress-induced autophagy in B cell lines and primary CLL cells

**Figure 3.**
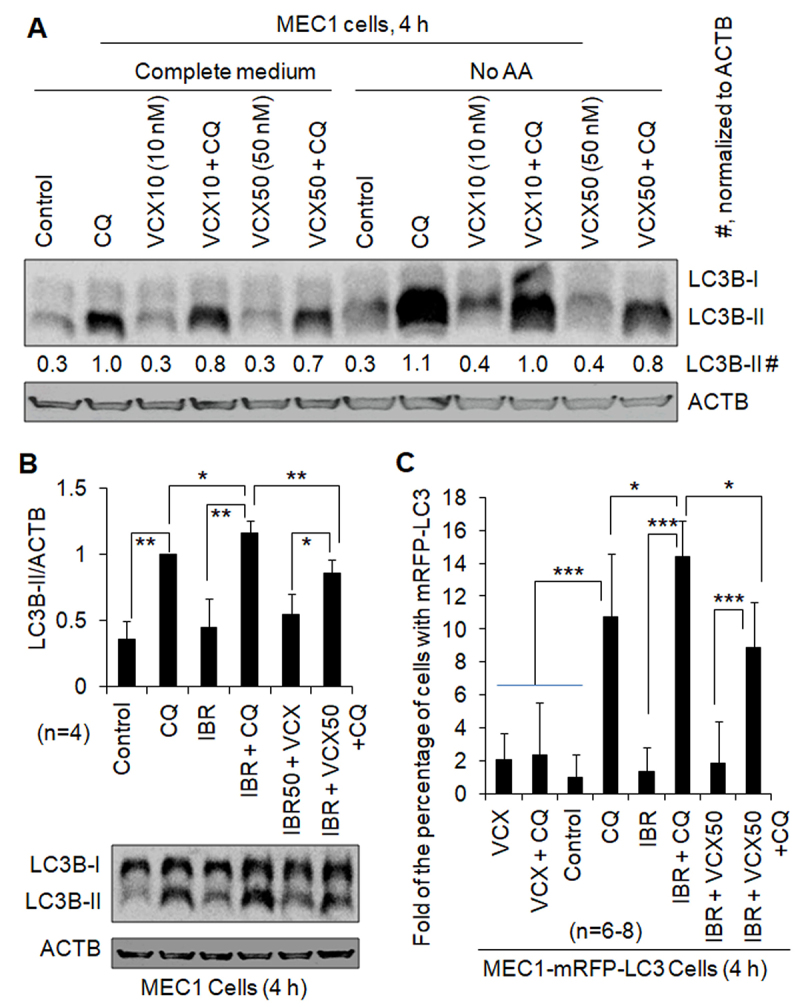
The effect of venetoclax on autophagy induced by starvation or ibrutinib. Autophagy was measured by western blotting the autophagy marker protein LC3-II (**A, B**) and quantifying the percentage of cells with mRFP-LC3 puncta (**C**) in the absence and presence of the lysosomal inhibitor chloroquine (CQ, 20 µM). ACTB was used as a loading control. IBR, ibrutinib (2.5 µM); VCX, venetoclax; VCX10, 10 nM VCX; VCX50, 50 nM VCX. (**A**) VCX regulated autophagy induced by starvation of amino acids (No AA) in MEC1 cells. (**B**) VCX regulated autophagy in the presence of IBR in MEC1 cells. (**C**) VCX regulated autophagy in the presence of IBR in MEC1-mRFP-LC3 cells. The data in Figure 1B were included in this figure for comparison. Since the percentage of cells with mRFP-LC3 puncta was less than 10%, the results were demonstrated by “Fold of the percentage of cells with mRFP-LC3 puncta”. At least 30 MEC1-mRFP-LC3 cells were counted per field under a fluorescent microscope. * *p*<0.05; ** *p*<0.01; *** *p*<0.001.

Under severe stress, such as starvation or drug treatment, autophagy can be induced higher than basal autophagy [12-14]. To investigate whether VCX can also inhibit stress-induced autophagy, we treated B cell lines and primary CLL cells with amino acid starvation (No AA) or IBR, an inhibitor of BTK. In MEC1 cells, the addition of CQ increased the levels of LC3B-II by at least 230% in the absence and presence of No AA, respectively (Fig. 3A), suggesting the existence of functional autophagy under the conditions without and with No AA. VCX at 10 nM and 50 nM reduced the levels of LC3B-II protein in the presence of CQ by 25% and 43%, respectively, without No AA, and by 9% and 27%, respectively, with No AA (Fig. 3A), supporting the concept that VCX inhibits starvation-induced autophagy. When MEC1 cells were treated with IBR, the level of LC3B-II protein and the percentage of cells with mRFP-LC3 puncta were not significantly changed in the absence of CQ but increased by 20% and 34% in the presence of CQ, respectively, and LC3B-II level and the percentage of cells with mRFP-LC3 puncta in the presence of IBR and CQ were reduced by 25% and 38%, respectively, by VCX (Fig. 3B, C). Similar phenomena were also observed in primary CLL cells. IBR treatment increased the levels of LC3B-II protein in the presence of CQ by 20%, 120%, and 10% in Patients #1, #3, and #4, respectively; in contrast, the levels of LC3B-II in the absence of CQ were not significantly changed (Fig. 2). Furthermore, the levels of LC3B-II in the presence of IBR and CQ were reduced by 27% and 45% by the addition of VCX, in Patients #3 and #4, respectively (Fig. 2B). Combined, these results indicate that IBR increases autophagy and VCX inhibits IBR-induced autophagy.

### VCX reduces the level of ATG12 protein

Our previous study demonstrated that ERBB2 promotes autophagy by upregulating the essential autophagy pathway protein, ATG12 (autophagy-related 12), in breast cancer cells [18]. To investigate the mechanism for VCX inhibition of autophagy, we measured levels of the ATG proteins, BECN1 (beclin 1) and ATG12, following VCX treatment in different types of B cell lines and primary CLL cells. In agreement with a previous study [15], VCX treatment led to a reduction of > 30% in BCL2 protein levels in MEC1, BJAB, CTL B cells, and primary CLL cells derived from 4 independent patients (Fig. S1). Interestingly, VCX treatment decreased ATG12 protein levels by > 20%; in contrast, BECN1 protein levels were not significantly changed (Fig. S1). These data support that VCX inhibition of autophagy at least correlates with the reduction of ATG12 protein.

### VCX sensitizes CLL cells to cell death

Autophagy mostly plays a pro-cell survival role. To investigate whether VCX inhibition of autophagy can sensitize CLL cells to cell death, we evaluated the effect of VCX on cell death induced by amino acid deprivation (No AA) or IBR treatment in primary CLL cells. The addition of VCX or CQ, another autophagy inhibitor, increased total cell death induced by No AA-induced by > 45% and by IBR by > 130% in primary CLL cells from Patient #3 and Patient #4, respectively (Fig. S2A). Furthermore, the combination of VCX and IBR greatly enhanced apoptosis compared to VCX or IBR alone in primary CLL cells from Patients #5 and #6 (Fig. S2B). Since VCX inhibited autophagy induced by No AA or IBR (Figs. 2B & 3), VCX could increase cell death at least partially by antagonizing the pro-cell survival mechanism of autophagy in B cell-derived cell lines and CLL.

## Discussion

VCX is a highly selective BCL2 inhibitor that is approved by the Food and Drug Administration (FDA) for the treatment of CLL and acute myeloid leukemia (AML) [19]. VCX is known to induce apoptosis, whereas other mechanisms associated with its death-causing role are still largely unknown. In this study, we demonstrated that VCX is a novel autophagy inhibitor in B cell lines and primary CLL cells, correlating with increased cell death in CLL cells.

BCL2 inhibition of autophagy [6,20] suggests that VCX may promote autophagy, and this hypothesis was supported by the conclusions from two reports [15,16]. However, in these studies, autophagic flux was not measured by quantifying the LC3-II protein levels or LC3 puncta in the absence and presence of a lysosomal inhibitor. Since autophagy plays a pro-cell survival role when it is in its “Goldilocks Zone” and can cause autophagy-induced cell death (AuICD) when over-enhanced [6,8], we initially hypothesized that VCX would induce AuICD. However, the results from the present study show that VCX inhibits autophagy.

It has been demonstrated that, in CLL cells, autophagy at its basal level can be increased by mTORC1 (mammalian target of rapamycin complex 1) inhibitors [12,13,21], the CDK (cyclin-dependent kinase) inhibitor, flavopiridol [12], and the nucleoside analog 8-Cl-Ado [14], in CLL cells. In contrast, autophagy in CLL cells is inhibited by the DNA damaging antitumor agent chlorambucil [12], the histone deacetylase (HDAC) inhibitors chidamide [9] and MGCD0103 [22], the small-molecule sirtuin inhibitor Tenovin-6 (Tnv-6) [13,21], the plant-derived sesquiterpene α-bisabolol (α-BSB) [23], and the microRNA (miRNA) miR-130a [24]. In this study, we report that autophagy in CLL cells can be inhibited by the BCL2 inhibitor VCX. These studies support that basal autophagy in CLL cells can either be promoted or be inhibited depending upon the context.

Since BCL2 inhibits autophagy and its inhibitors are expected to promote autophagy [6,20], our finding indicates that VCX inhibits autophagy via a mechanism different from its effect on BCL2, which will be a focus of our future studies, including the detailed studies of VCX’s effects on the expression of *ATG12* and other autophagy-related (ATG) genes.

We also demonstrated that IBR increased autophagy in B cell lines and primary CLL cells. VCX can inhibit autophagy induced by amino acid starvation or IBR and sensitize primary CLL cells to death caused by starvation or IBR. A previous study reported that autophagy promotes cell survival in CLL [12]. Our findings that VCX is a novel autophagy inhibitor and sensitizes CLL cells to IBR provide a potential new mechanism that at least partially explains the marked antitumor activity of VCX seen on combination therapies in the clinic [25,26].

## Materials and Methods

### Reagents and Antibodies

Trypan blue solution (T8154), sodium orthovanadate (Na_3_VO_4_) (S6508), 3-methyladenine (3-MA; M9281), chloroquine diphosphate (CQ; C6628), and phosphatase inhibitor cocktails 2 and 3 (P5726, P0044), NP40 (I8896), okadaic acid (O7885), aprotinin (A1153), pepstatin A (P5318), leupeptin (L2884), and phenylmethanesulfonyl fluoride (PMSF) (93482) were purchased from Sigma-Aldrich, Lipofectamine^TM^ 2000 transfection reagent (11668019) and G418 disulfate (J63871.AB) from Thermo Fisher Scientific, and protease inhibitor cocktail (11 836 153 001) from Roche Diagnostics.

Primary antibodies: anti-BCL2 (15071), anti-BECN1 (3495), anti-ATG12 (4180), anti-LC3B (2775S), and anti-SQSTM1/p62 (D1Q5S) (39749), were purchased from Cell Signaling Technology, and anti-ACTB/actin beta from Sigma-Aldrich (A3853). Secondary antibodies: goat anti-rabbit IgG (H^+^L)-HRP conjugate (170–6515) and goat anti-mouse IgG (H^+^L)-HRP conjugate (170–6516) were obtained from Bio-Rad Laboratories. EBSS Medium for AA (amino acids and serum) starvation (SH30029.02) was purchased from HyClone Laboratories Inc. (Logan, UT, USA).

### Cell Lines and Primary CLL Cells from Patients

The human CLL cell line MEC1, the human malignant EBV (Epstein-Bar virus)-negative Burkitt-like lymphoma cell line BJAB, an EBV-immortalized normal B cell lymphoblastic cell line (CTL B cells), and primary CLL cells were incubated or maintained in Gibco RPMI 1640 medium (Thermo Fisher Scientific, 11875093) supplemented with 10% fetal bovine serum (FBS), and 100 units of penicillin per mL plus 100 µg of streptomycin per mL (Life Technologies, 15140–122), in a humidified incubator at 5% CO_2_, 37°C. CTL B cells were obtained from Coriell Institute (Camden, NJ, USA). Primary CLL cells were isolated from the peripheral blood mononuclear cells (PBMCs) of human patients at the Manitoba CLL Clinic by Manitoba Tumour Bank. Primary CLL cells from 6 patients were used in this study, including Patient # 1 (FISH: del 13; IGVH: ND (not decided); TP53: ND), Patient # 2 (FISH: ND; IGVH: ND; TP53: ND), Patient # 3 (FISH: Tri 12; IGVH: U (unmutated); TP53: Neg (negative)), Patient # 4 (FISH: ND; IGVH: M (mutated); TP53: ND), Patient # 5 (FISH: ND; IGVH: ND; TP53: ND), and Patient # 6 (FISH: del 11; IGVH: ND; TP53: Neg).

### Western Blot Analysis

Western blot was described in our previous publication [27]. Total cell lysate (TCL) was obtained by lysis of cells with NP40 protein lysis buffer containing 0.5% NP40, 250 mM NaCl, 50 mM Tris HCl, pH 7.4, 50 mM NaF, 15 mM sodium pyrophosphate, 1 mM glycerophosphate, 1 mM Na_3_VO_4_, 500 nM okadaic acid, 20 μg/ml aprotinin, 0.7 μg/ml pepstatin A, 5 μg/ml leupeptin, 1 mM PMSF, with the addition of protease inhibitor cocktail and phosphatase inhibitor cocktails 2 and 3. The principle of the linear range of detection was followed for western blotting. Western blot data were generated by using 10–50 µg total proteins using ACTB as the housekeeping gene protein. We used the ImageJ program to quantify the intensities of protein bands.

### Measurement of Autophagy

Autophagy was measured by western blotting the autophagy marker protein LC3B-II (or the autophagy substrate protein SQSTM1/p62) and fluorescent microscopy measurement of autophagosomes/autolysosomes as described in our previous publications [27–29]. During autophagy, the cytosolic form of LC3, LC3-I, is converted to its lipidated form, LC3-II, on the double membranes of an autophagosome. Then, LC3-II on the inner membrane of the autophagosome will be degraded in the autolysosome, whereas LC3-II on the outer membrane of the autophagosome will be delipidated by ATG4 to become LC3-I when it is relocated on the autolysosome which is generated from the autophagosome. Since autophagy is a dynamic process, functional autophagy must be determined by evaluating autophagic flux. Autophagic flux can be measured by quantifying the amount of LC3-II or an autophagy substrate (e.g., SQSTM1/p62) in the absence and presence of a lysosomal inhibitor via western blot. A high level of LC3-II (or SQSTM1/p62) in the presence of a lysosomal inhibitor compared to that in the absence of such an inhibitor indicates a positive autophagic flux and, therefore, functional autophagy. Under functional autophagy, levels of autophagy can be determined by comparing the LC3-II (or SQSTM1/p62) levels in the presence of a lysosomal inhibitor. This study used the lysosomal inhibitor chloroquine (CQ) to measure autophagic flux. It should be noted that, although evaluating the levels of LC3-II is essential for autophagy measurement, especially by western blot under the conditions when enough amounts of cellular proteins are available, the levels of SQSTM1/p62 may not reflect autophagy changes under some conditions since SQSTM1/p62 is just one type of autophagy substrates and its expression can be affected by autophagic degradation, transcriptional upregulation, and the availability of lysosome-derived amino acids [30]. In this study, western blotting of SQSTM1/p62 was only used as a supporting method for autophagy measurement in some cell types. By fluorescent microscopy, autophagy was also measured by quantifying the percentage of cells with autophagosomes or autolysosomes. The pmRFP-LC3B plasmid (Addgene, 21,075; deposited by Tamotsu Yoshimori) [31] was transfected into MEC1 cells with Lipofectamine^TM^ 2000, and a stable cell line (MEC1-mRFP-LC3) was developed by treating a pool of transfected cells with G418 for over two months. Cells with and without treatment were attached to a microscope slide on a Cytospin centrifuge, as described in our previous publication [18]. Then, the expression of LC3 (red color) was observed with a fluorescence microscope. The diffused red fluorescent color represents LC3-I, and the mRFP-LC3 red puncta represent autophagosomes or autolysosomes. Autophagy was quantified by counting the percentage of cells with mRFP-LC3 puncta. CQ was used to measure autophagic flux.

### Analysis of Total Cell Death by Trypan Blue Exclusion Assay

Total cell death was calculated based on the number of live cells before and after treatment by considering the lysed (dead) and non-lysed (live and dead) cells. Live cells were counted on a DeNOVIX CellDrop FL fluorescence cell counter following trypan blue staining, as described in our previous study [28]. The formula for total cell death calculation is expressed as follows:

Total cell death (%) = [(A-B)/A] x 100

A: Number of live cells/mL before treatment

B: Number of live cells/mL after treatment

### Measurement of Apoptosis by Flow Cytometry

Apoptosis was measured as described in our previous study [32]. CLL cells were treated in serum-free hybridoma media (Life Technologies, Carlsbad, CA) in 96-well plates with 1.25 nM venetoclax and/or 2.5 µM IBR (both from Selleckchem, Houston, TX) for 72 h at 37°C and 5% CO_2_ in a humidified atmosphere. DMSO alone was used as a control. Cell death was determined by flow cytometry using annexin V-FITC and 7-aminoactinomycin D (7AAD) (both from BD Biosciences, San Jose, CA). Cells were stained with annexin V-FITC and 7AAD for 15 minutes and analyzed using a NovoCyte flow cytometer (ACEA Biosciences, San Diego, CA).

## Statistical Analysis

Data in this study represent at least two independent experiments. Four replicate experiments were performed for the total cell death assay. Data were presented as means ± standard deviation (SD) (n≥3). The Student *t*-test with two-tailed distribution and unequal variances was performed for statistical analysis. A value of *p* < 0.05 is considered to be statistically significant. *p < 0.5; **p < 0.01; ***p < 0.001.

## Supplementary Material

Supplemental Material
