# Supplementary material for: Venetoclax inhibits autophagy in chronic lymphocytic leukemia cells
Source: Autophagy Rep. 2023 Feb 7;2(1):2169518. doi: 10.1080/27694127.2023.2169518 (PMC12042475; doi:10.1080/27694127.2023.2169518)
Supplement: Supplemental Material [file KAUO_A_2169518_SM7252.zip › Revised Suppl VCX autophagy (1).pptx]

## Slide 1
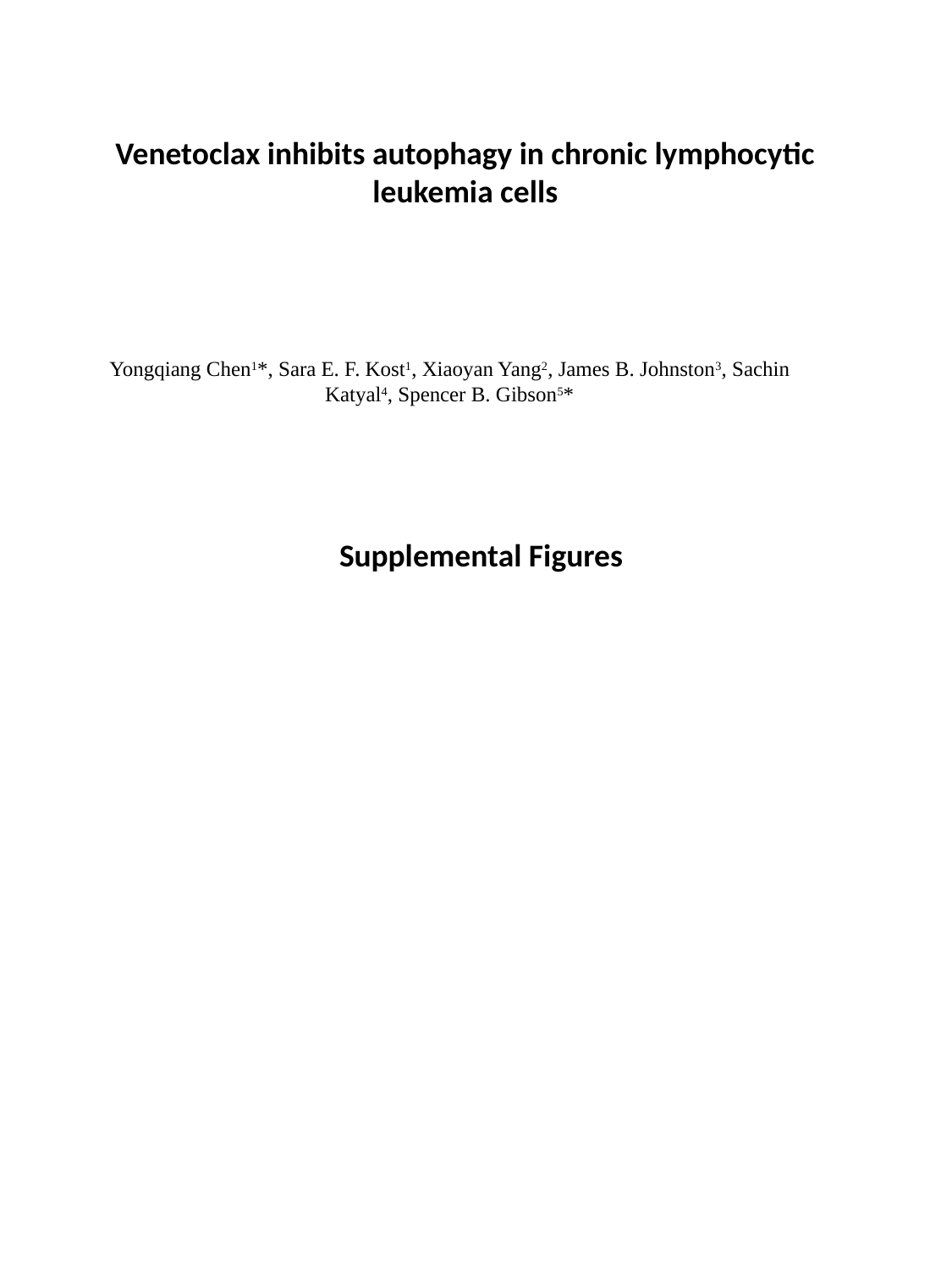

Venetoclax inhibits autophagy in chronic lymphocytic leukemia cells
Yongqiang Chen1*, Sara E. F. Kost1, Xiaoyan Yang2, James B. Johnston3, Sachin Katyal4, Spencer B. Gibson5*
Supplemental Figures

## Slide 2
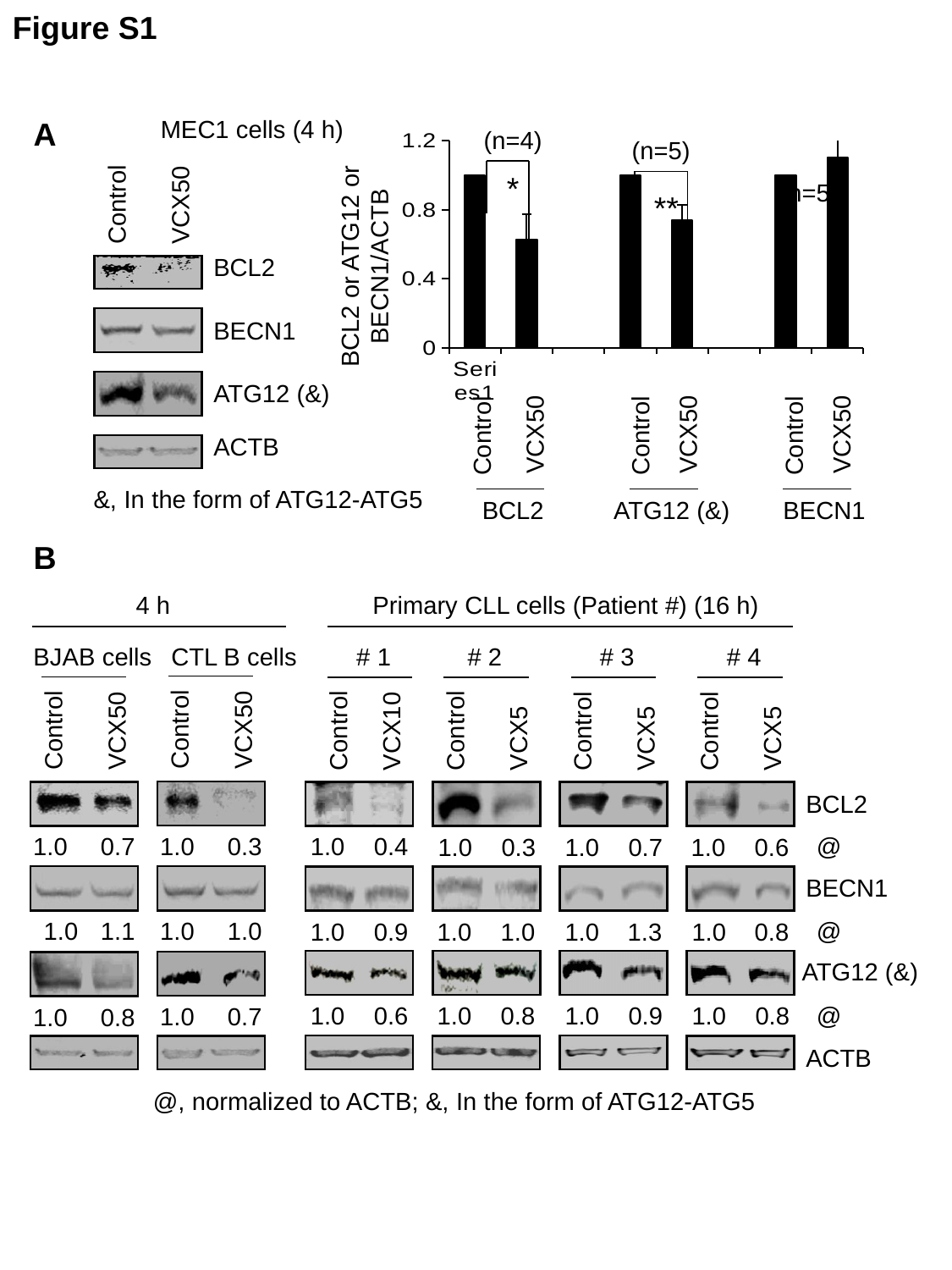

Figure S1
A
MEC1 cells (4 h)
VCX50
Control
BCL2
BECN1
ATG12 (&)
ACTB
(n=4)
### Chart
| Category | |
|---|---|
| | 1.0 |
| | 0.6250000000000021 |
| | None |
| | 1.0 |
| | 0.740000000000002 |
| | None |
| | 1.0 |
| | 1.1 |(n=5)
*
(n=5)
**
BCL2 or ATG12 or BECN1/ACTB
VCX50
VCX50
VCX50
Control
Control
Control
BCL2
BECN1
ATG12 (&)
 &, In the form of ATG12-ATG5
B
Primary CLL cells (Patient #) (16 h)
BJAB cells
CTL B cells
 # 1
 # 2
 # 3
Control
VCX5
1.0
0.7
1.0
1.3
1.0
0.9
 # 4
Control
VCX5
1.0
0.6
1.0
0.8
1.0
0.8
Control
VCX50
Control
VCX50
Control
VCX10
Control
VCX5
BCL2
1.0
0.3
1.0
0.4
1.0
0.7
@
1.0
0.3
BECN1
1.0
1.1
1.0
1.0
@
1.0
0.9
1.0
1.0
ATG12 (&)
1.0
0.6
1.0
0.8
@
1.0
0.7
1.0
0.8
ACTB
@, normalized to ACTB; &, In the form of ATG12-ATG5
4 h

## Slide 3
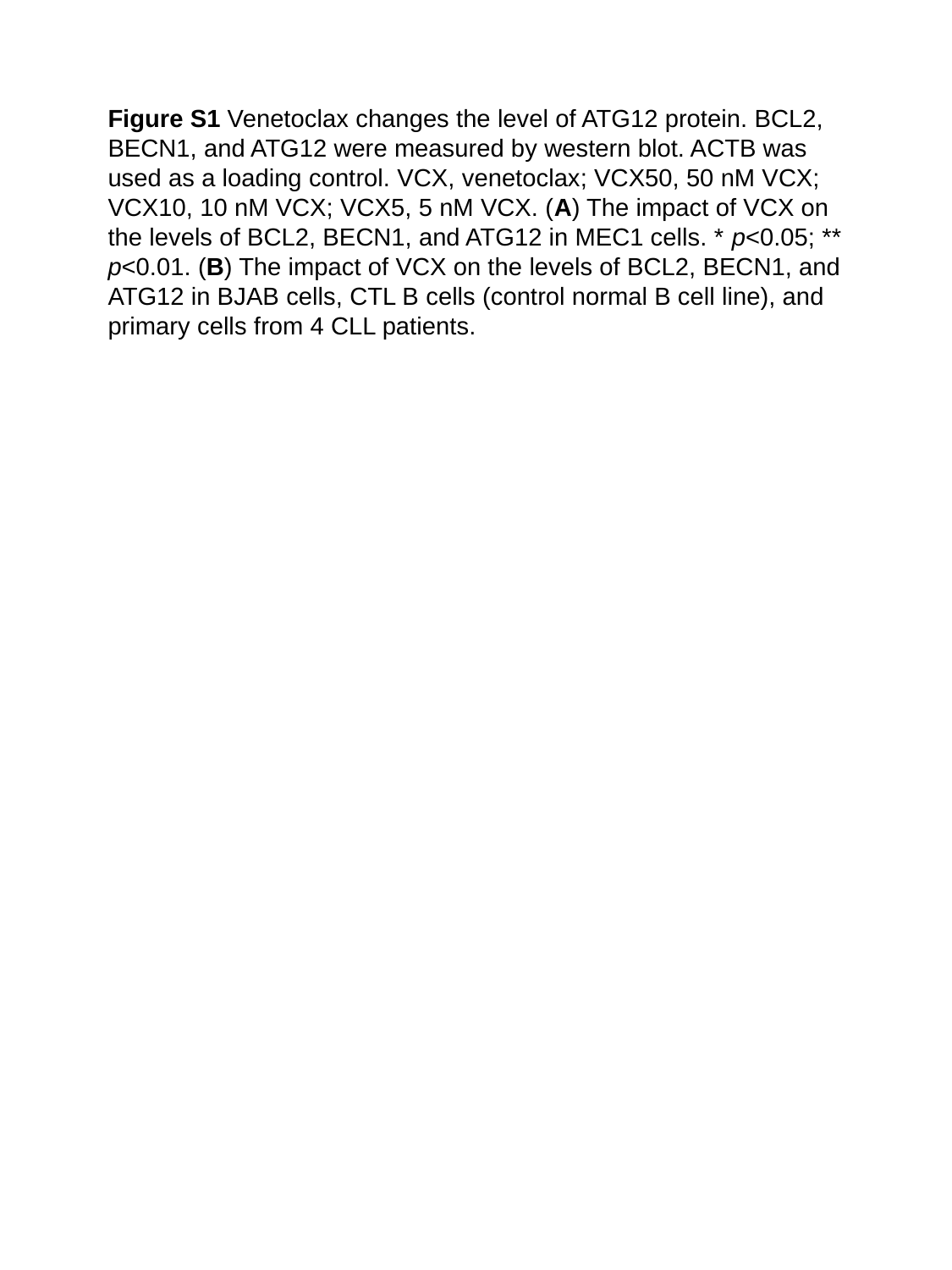

Figure S1 Venetoclax changes the level of ATG12 protein. BCL2, BECN1, and ATG12 were measured by western blot. ACTB was used as a loading control. VCX, venetoclax; VCX50, 50 nM VCX; VCX10, 10 nM VCX; VCX5, 5 nM VCX. (A) The impact of VCX on the levels of BCL2, BECN1, and ATG12 in MEC1 cells. * p<0.05; ** p<0.01. (B) The impact of VCX on the levels of BCL2, BECN1, and ATG12 in BJAB cells, CTL B cells (control normal B cell line), and primary cells from 4 CLL patients.

## Slide 4
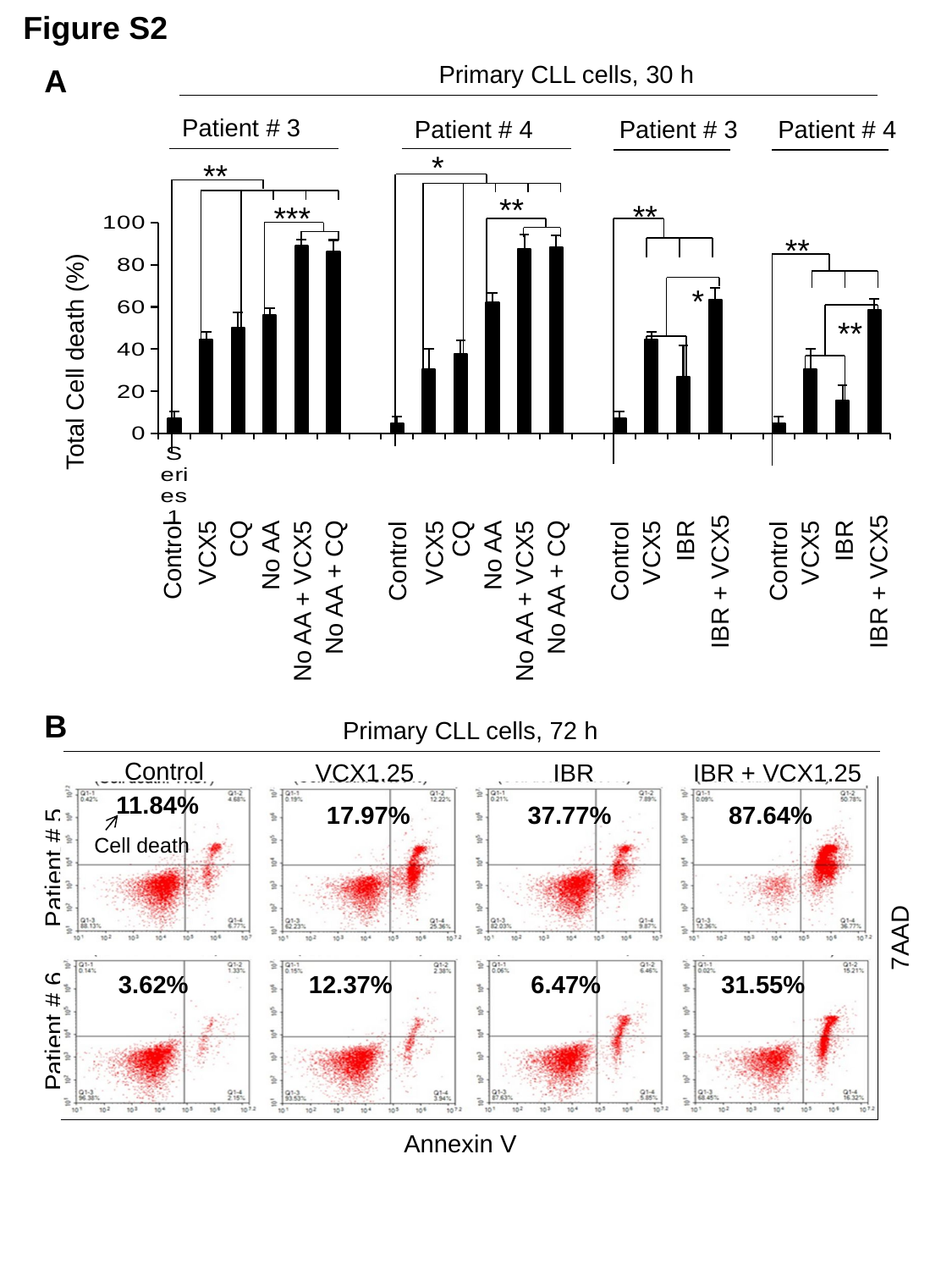

Figure S2
Primary CLL cells, 30 h
A
Patient # 3
Patient # 4
Patient # 3
Patient # 4
*
**
**
***
**
*
### Chart
| Category | |
|---|---|
| | 7.111110999999984 |
| | 44.5 |
| | 50.27778 |
| | 56.05556 |
| | 88.83332999999999 |
| | 86.38888999999999 |
| | None |
| | 5.0 |
| | 30.66667 |
| | 37.83333000000001 |
| | 62.0 |
| | 87.38888999999999 |
| | 88.11111 |
| | None |
| | 7.111110999999984 |
| | 44.5 |
| | 26.94443999999991 |
| | 63.55556 |
| | None |
| | 5.0 |
| | 30.66667 |
| | 15.555560000000026 |
| | 58.55556 |**
**
Total Cell death (%)
CQ
CQ
IBR
IBR
No AA
No AA
Control
Control
Control
Control
VCX5
VCX5
VCX5
VCX5
IBR + VCX5
IBR + VCX5
No AA + CQ
No AA + CQ
No AA + VCX5
No AA + VCX5
B
Primary CLL cells, 72 h
Control
VCX1.25
IBR
IBR + VCX1.25
Cell death
Patient # 5
7AAD
Patient # 6
Annexin V
11.84%
17.97%
37.77%
87.64%
3.62%
12.37%
6.47%
31.55%

## Slide 5
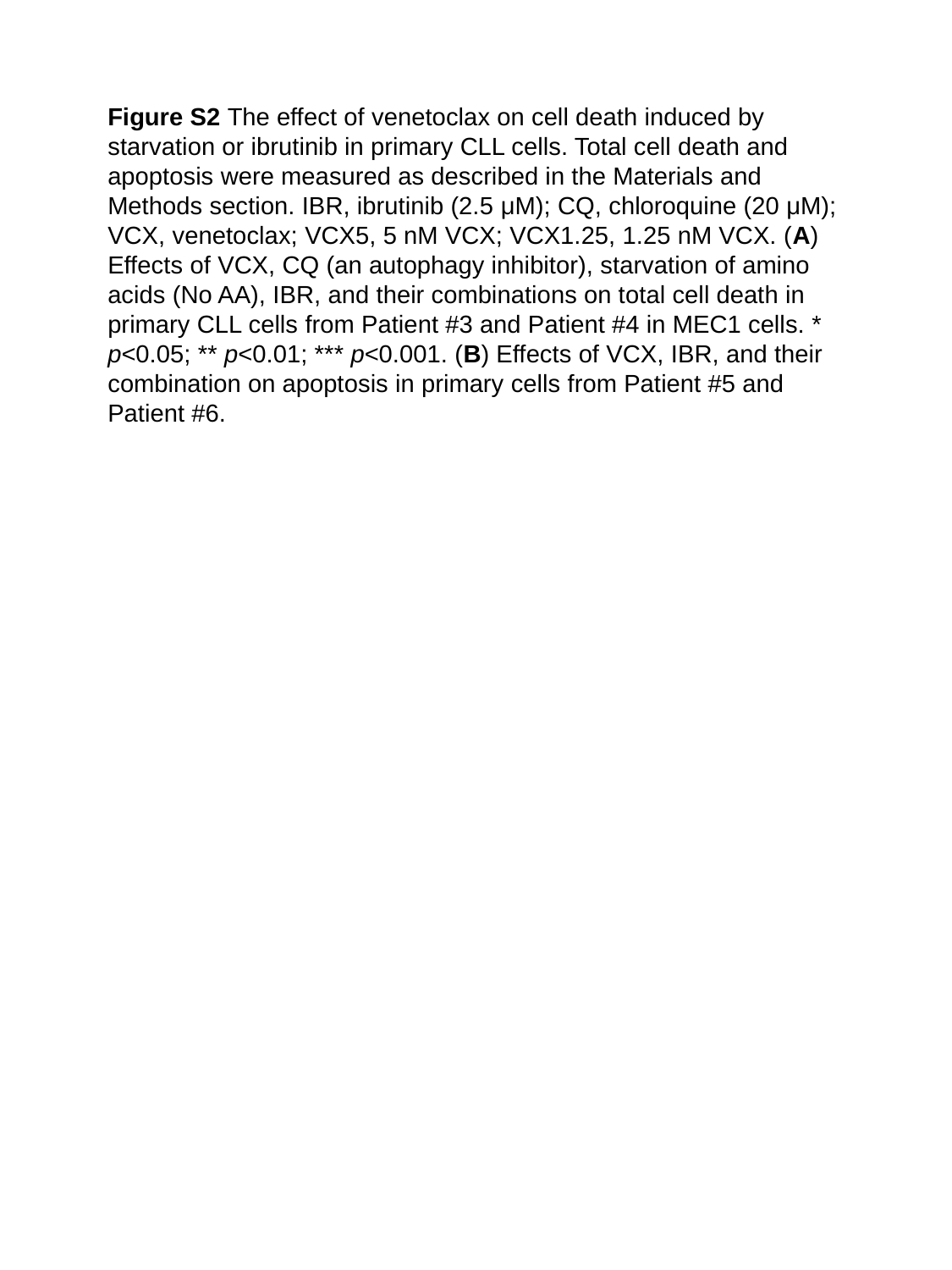

Figure S2 The effect of venetoclax on cell death induced by starvation or ibrutinib in primary CLL cells. Total cell death and apoptosis were measured as described in the Materials and Methods section. IBR, ibrutinib (2.5 μM); CQ, chloroquine (20 μM); VCX, venetoclax; VCX5, 5 nM VCX; VCX1.25, 1.25 nM VCX. (A) Effects of VCX, CQ (an autophagy inhibitor), starvation of amino acids (No AA), IBR, and their combinations on total cell death in primary CLL cells from Patient #3 and Patient #4 in MEC1 cells. * p<0.05; ** p<0.01; *** p<0.001. (B) Effects of VCX, IBR, and their combination on apoptosis in primary cells from Patient #5 and Patient #6.
