# Supplementary material for: Venetoclax inhibits autophagy in chronic lymphocytic leukemia cells
Source: Autophagy Rep. 2023 Feb 7;2(1):2169518. doi: 10.1080/27694127.2023.2169518 (PMC12042475; doi:10.1080/27694127.2023.2169518)
Supplement: Supplemental Material [file KAUO_A_2169518_SM7252.zip › Suppl figures for VCX man (1).pptx]

## Slide 1
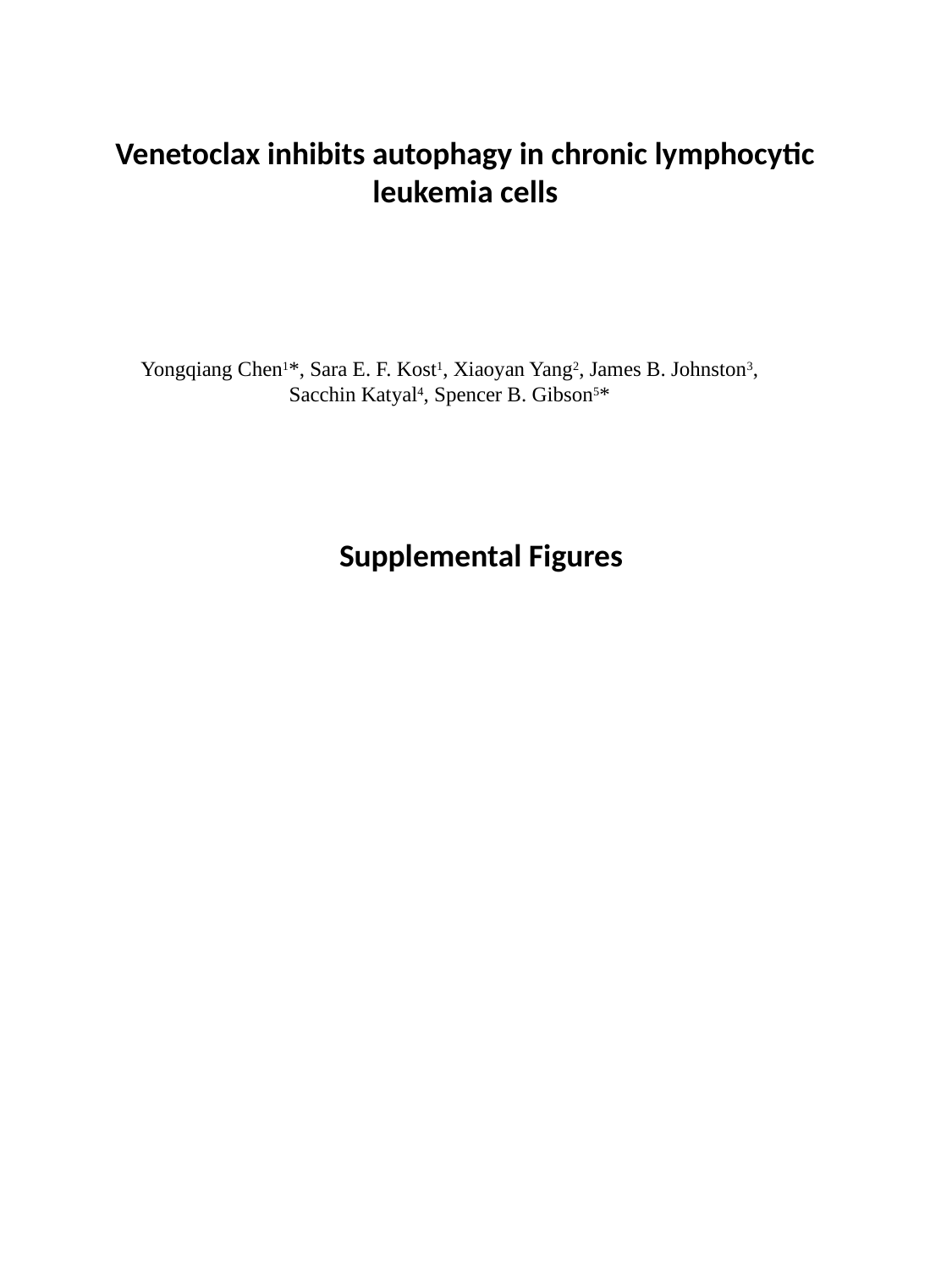

Venetoclax inhibits autophagy in chronic lymphocytic leukemia cells
Yongqiang Chen1*, Sara E. F. Kost1, Xiaoyan Yang2, James B. Johnston3, Sacchin Katyal4, Spencer B. Gibson5*
Supplemental Figures

## Slide 2
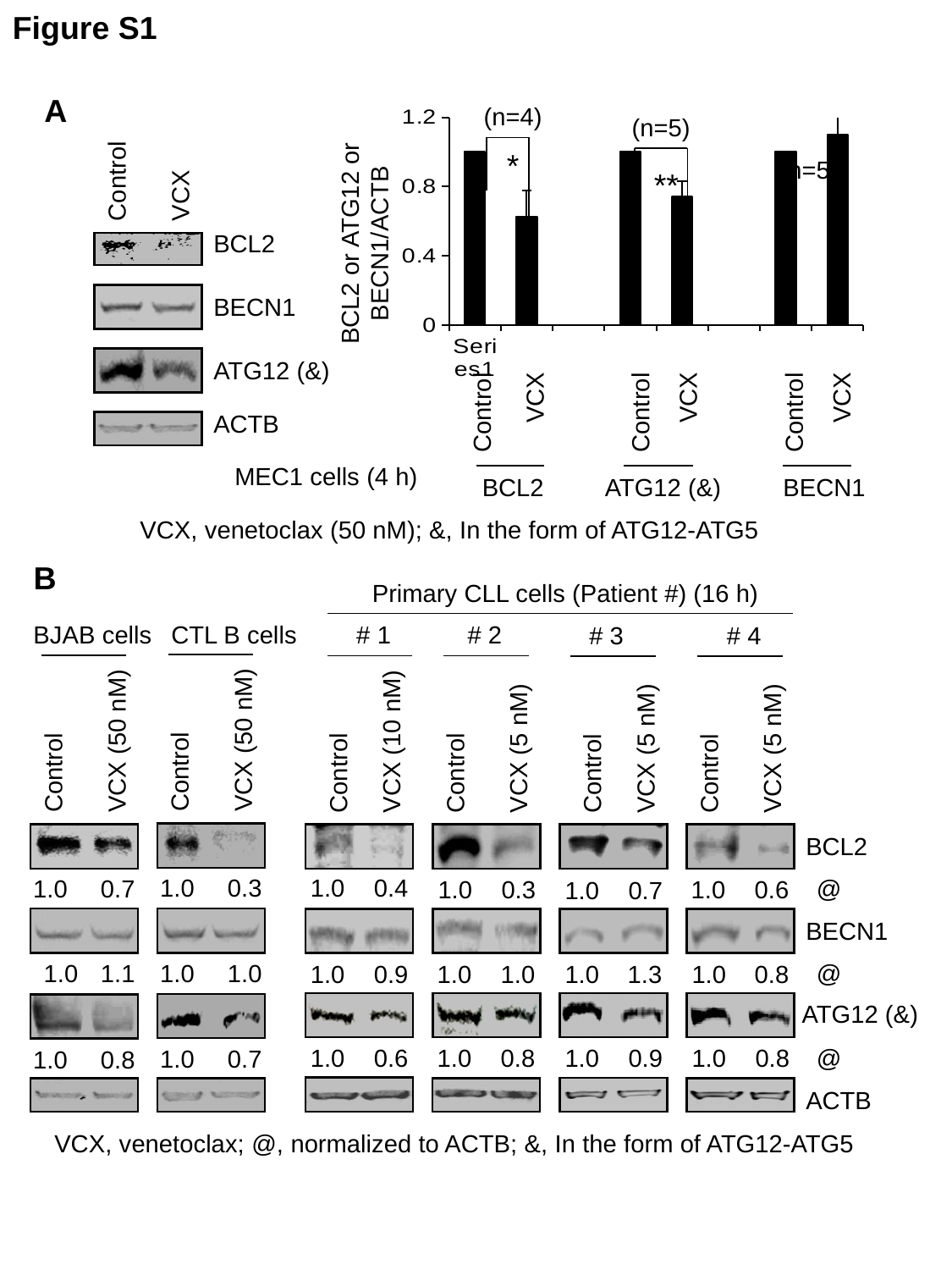

Figure S1
A
(n=4)
### Chart
| Category | |
|---|---|
| | 1.0 |
| | 0.6250000000000016 |
| | None |
| | 1.0 |
| | 0.7400000000000014 |
| | None |
| | 1.0 |
| | 1.1 |(n=5)
*
(n=5)
**
BCL2 or ATG12 or BECN1/ACTB
VCX
VCX
VCX
Control
Control
Control
BCL2
ATG12 (&)
BECN1
Control
VCX
BCL2
BECN1
ATG12 (&)
ACTB
MEC1 cells (4 h)
VCX, venetoclax (50 nM); &, In the form of ATG12-ATG5
B
Primary CLL cells (Patient #) (16 h)
BJAB cells
CTL B cells
 # 1
 # 2
 # 3
VCX (5 nM)
Control
1.0
0.7
1.0
1.3
1.0
0.9
 # 4
VCX (5 nM)
Control
1.0
0.6
1.0
0.8
1.0
0.8
VCX (10 nM)
VCX (50 nM)
VCX (50 nM)
VCX (5 nM)
Control
Control
Control
Control
BCL2
1.0
0.3
1.0
0.4
1.0
0.7
@
1.0
0.3
BECN1
1.0
1.1
1.0
1.0
@
1.0
0.9
1.0
1.0
ATG12 (&)
1.0
0.6
1.0
0.8
@
1.0
0.7
1.0
0.8
ACTB
VCX, venetoclax; @, normalized to ACTB; &, In the form of ATG12-ATG5

## Slide 3
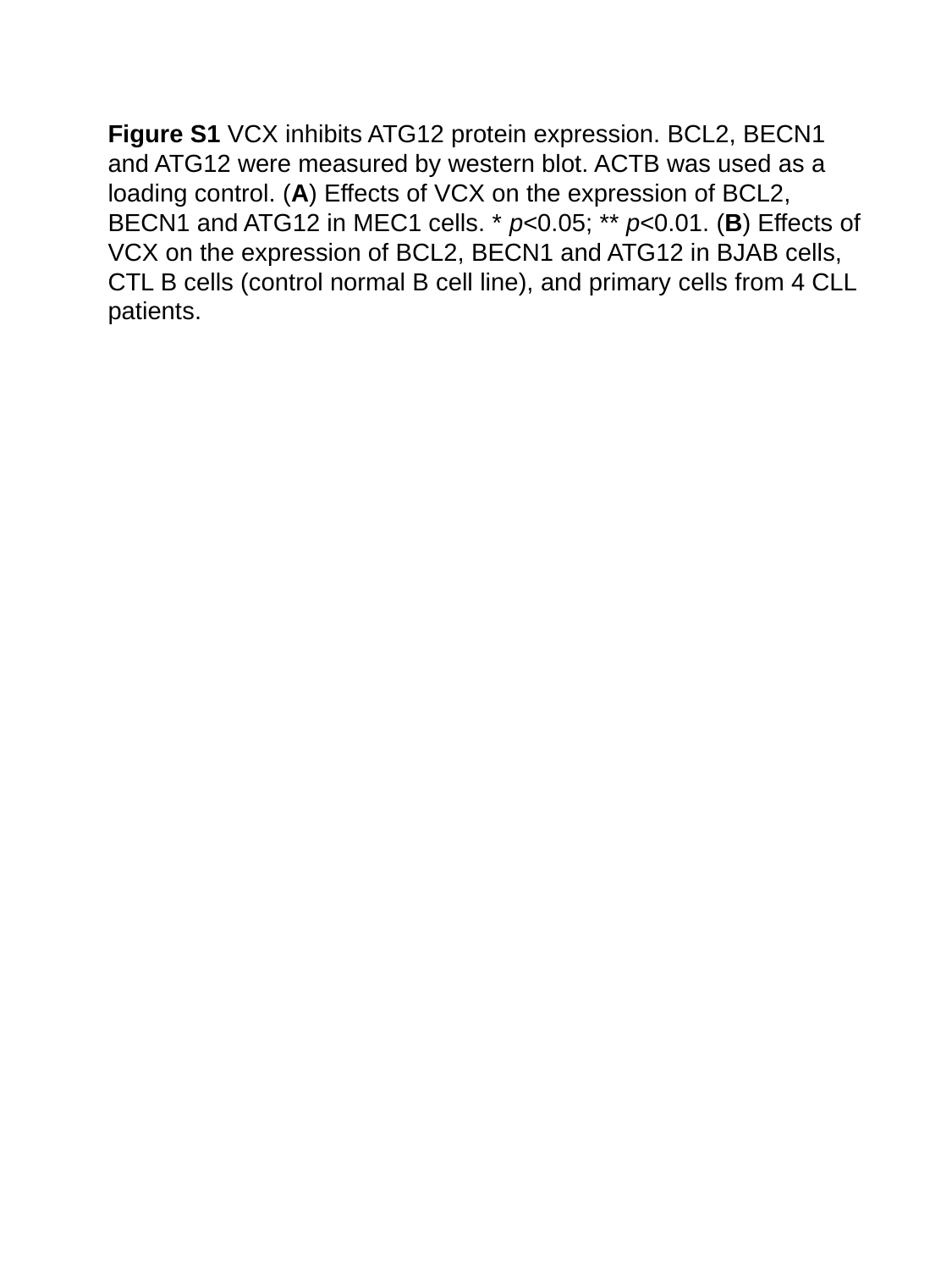

Figure S1 VCX inhibits ATG12 protein expression. BCL2, BECN1 and ATG12 were measured by western blot. ACTB was used as a loading control. (A) Effects of VCX on the expression of BCL2, BECN1 and ATG12 in MEC1 cells. * p<0.05; ** p<0.01. (B) Effects of VCX on the expression of BCL2, BECN1 and ATG12 in BJAB cells, CTL B cells (control normal B cell line), and primary cells from 4 CLL patients.

## Slide 4
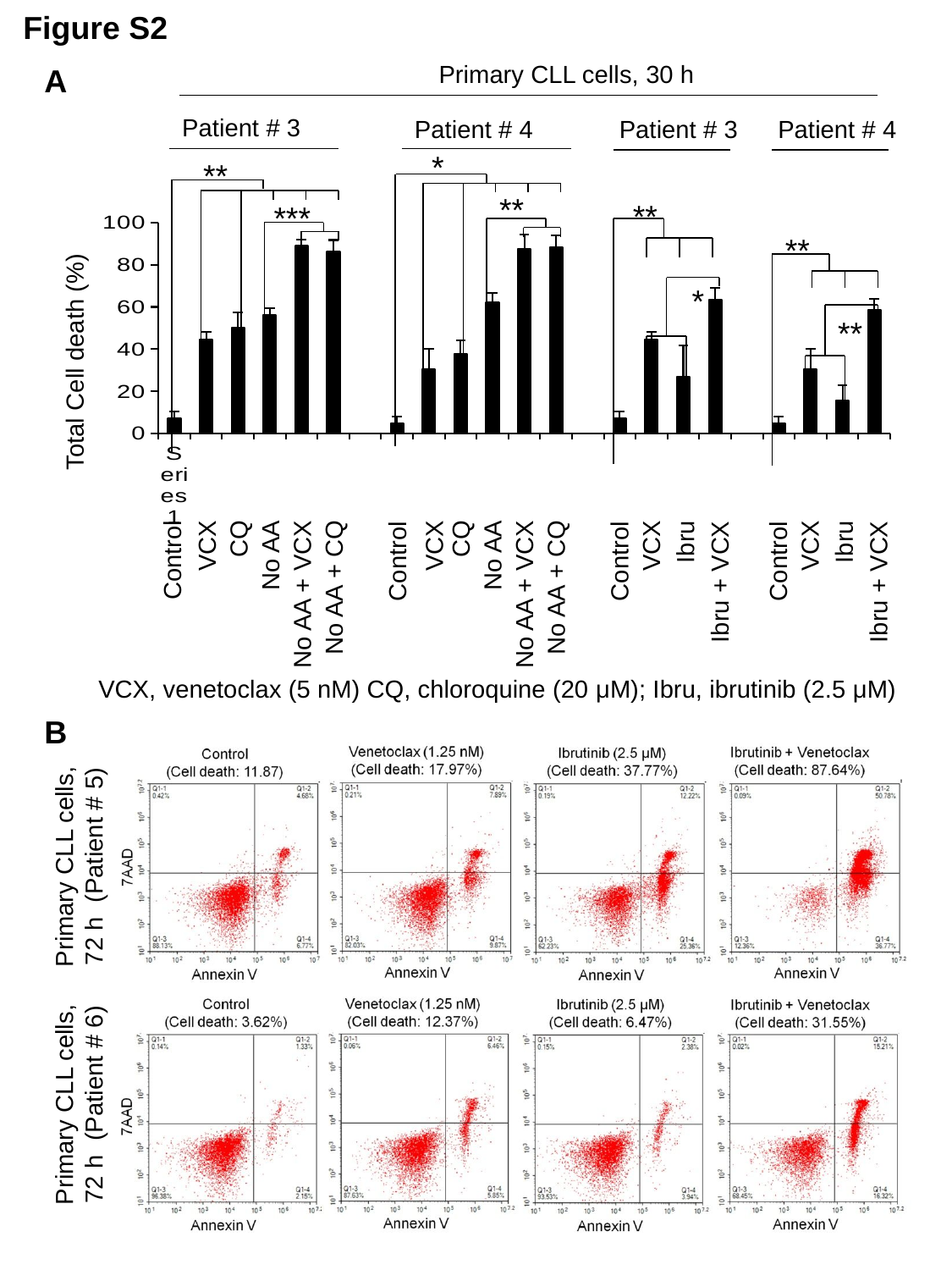

Figure S2
Primary CLL cells, 30 h
A
Patient # 3
Patient # 4
Patient # 3
Patient # 4
*
**
**
***
**
*
### Chart
| Category | |
|---|---|
| | 7.1111109999999895 |
| | 44.5 |
| | 50.27778 |
| | 56.05556 |
| | 88.83332999999999 |
| | 86.38888999999999 |
| | None |
| | 5.0 |
| | 30.66667 |
| | 37.83333000000001 |
| | 62.0 |
| | 87.38888999999999 |
| | 88.11111 |
| | None |
| | 7.1111109999999895 |
| | 44.5 |
| | 26.944439999999947 |
| | 63.55556 |
| | None |
| | 5.0 |
| | 30.66667 |
| | 15.555560000000016 |
| | 58.55556 |**
**
Total Cell death (%)
CQ
CQ
Ibru
Ibru
VCX
VCX
VCX
VCX
No AA
No AA
Control
Control
Control
Control
Ibru + VCX
Ibru + VCX
No AA + CQ
No AA + CQ
No AA + VCX
No AA + VCX
VCX, venetoclax (5 nM) CQ, chloroquine (20 μM); Ibru, ibrutinib (2.5 μM)
B
Primary CLL cells, 72 h (Patient # 5)
Primary CLL cells, 72 h (Patient # 6)

## Slide 5
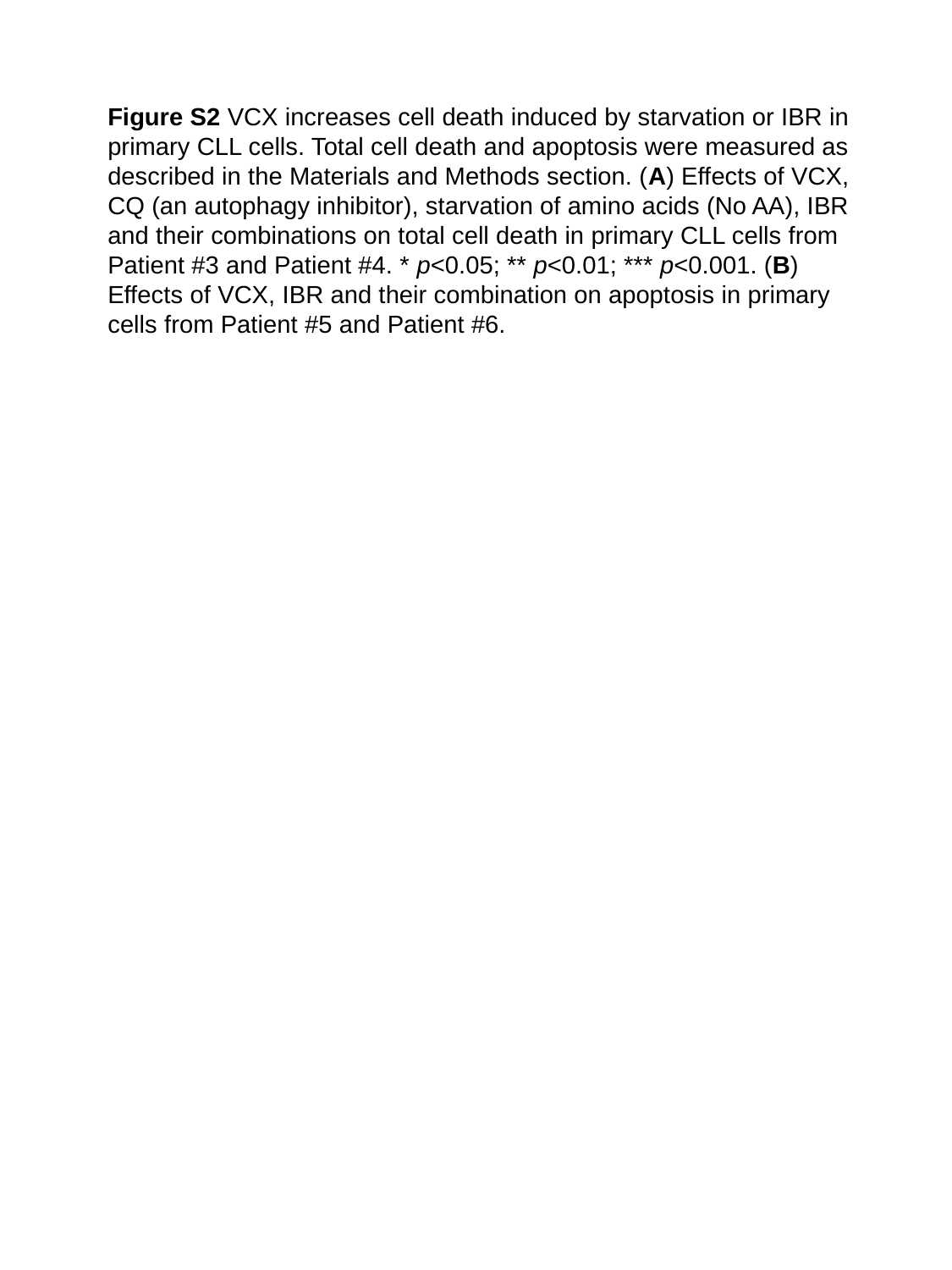

Figure S2 VCX increases cell death induced by starvation or IBR in primary CLL cells. Total cell death and apoptosis were measured as described in the Materials and Methods section. (A) Effects of VCX, CQ (an autophagy inhibitor), starvation of amino acids (No AA), IBR and their combinations on total cell death in primary CLL cells from Patient #3 and Patient #4. * p<0.05; ** p<0.01; *** p<0.001. (B) Effects of VCX, IBR and their combination on apoptosis in primary cells from Patient #5 and Patient #6.
